# Structure, Function and Inhibition of the Phosphoethanolamine Methyltransferases of the Human Malaria Parasites *Plasmodium vivax* and *Plasmodium knowlesi*

**DOI:** 10.1038/srep09064

**Published:** 2015-03-12

**Authors:** Aprajita Garg, Tiit Lukk, Vidya Kumar, Jae-Yeon Choi, Yoann Augagneur, Dennis R. Voelker, Satish Nair, Choukri Ben Mamoun

**Affiliations:** 1 Department of Internal Medicine, Yale University School of Medicine, New Haven CT, 06520 USA; 2 Department of Biochemistry, University of Illinois at Urbana-Champaign; 3 Basic Science Section, Department of Medicine, National Jewish Health, Denver, Colorado 80206; 4 Cornell High Energy Synchrotron Source, Cornell University

## Abstract

Phosphoethanolamine methyltransferases (PMTs) catalyze the three-step methylation of phosphoethanolamine to form phosphocholine, a critical step in the synthesis of phosphatidylcholine in a select number of eukaryotes including human malaria parasites, nematodes and plants. Genetic studies in the malaria parasite *Plasmodium falciparum* have shown that the methyltransferase PfPMT plays a critical function in parasite development and differentiation. The presence of PMT orthologs in other malaria parasites that infect humans and their absence in mammals make them ideal targets for the development of selective antimalarials with broad specificity against different *Plasmodium* species. Here we describe the X-ray structures and biochemical properties of PMT orthologs from *Plasmodium vivax* and *Plasmodium knowlesi* and show that both enzymes are inhibited by amodiaquine and NSC158011, two drugs with potent antimalarial activity. Metabolic studies in a yeast mutant that relies on PkPMT or PvPMT for survival demonstrated that these compounds inhibit phosphatidylcholine biosynthesis from ethanolamine. Our structural and functional data provide insights into the mechanism of catalysis and inhibition of PMT enzymes and set the stage for a better design of more specific and selective antimalarial drugs.

Of all parasitic infections, malaria, caused by *Plasmodium* species, remains the leading cause of deaths in humans. Four *Plasmodium* species *P. falciparum*, *P. vivax*, *P. ovale* and *P. malariae* commonly cause infection in humans with the first two species responsible for most clinical cases and fatalities^1^. Cases of infections caused by other *Plasmodium* species including *P. knowlesi,* which normally infect non-human primates, have also been reported^2^,^3^. These cases have raised concerns about the rapid adaptation of these parasites to humans and the presence of a mammalian reservoir that could make eradication a rather difficult task. In the absence of an effective, safe and easily deployable malaria vaccine, current efforts to eradicate malaria have focused on the development of drugs that target different stages of the parasite life cycle and particularly those that block intraerythrocytic development and malaria transmission^4^. However, most of these therapeutic efforts have been limited to *P. falciparum* due to the availability of an *in vitro* culture system and understandably to the high fatality rate caused by this parasite. Drugs developed for *P. falciparum* are subsequently evaluated against other human malaria parasites with limited success due to the evolutionary separation between the species, their different mechanisms of pathogenesis and distinct mechanisms of drug resistance. Novel therapies that target conserved metabolic pathways and cellular functions important for both asexual development and sexual differentiation in all human malaria parasites are thus needed to accomplish a successful eradication program.

Recent efforts aimed to complete the genome sequence and annotation of several *Plasmodium* species have helped identify genes and pathways conserved among different human malaria parasites^5^,^6^,^7^. Among these pathways, the metabolic routes for the synthesis of parasite phospholipids from host choline and serine have emerged as ideal targets because they include steps that are either absent, or different from those found in humans^8^.

Phosphatidylcholine (PC) is the major phospholipid constituent of the membranes of *Plasmodium* parasites and it plays an essential role in parasite development and survival^8^,^9^,^10^,^11^,^12^,^13^,^14^,^15^. Accordingly, drugs that target different critical steps in the biosynthesis of PC, or mimic its chemical structure, display potent antimalarial activity *in vitro* and *in vivo*, with some showing promising results in clinical trials for treatment of drug-resistant malaria^9^,^13^,^16^,^17^,^18^,^19^,^20^. Biochemical and genetic studies demonstrate two major non-redundant pathways (CDP-choline pathway and SDPM pathway) for the synthesis of PC in *Plasmodium*^21^. In *P. falciparum*, the unusual step in the synthesis of PC from ethanolamine is catalyzed by a monopartite methyltranferase, PfPMT, which transfers 3 methyl groups to the amine of phosphoethanolamine producing phosphocholine^13^,^22^,^23^. PfPMT is a defining member of a new class of methyltransferases found in a limited number of species including plants, nematodes and other *Plasmodium* species, but absent in mammals^21^. Interestingly, among *Plasmodium* species, only those infecting humans and other non-human primates express orthologs of PfPMT^21^. Deletion of *PfPMT* gene results in major developmental defects during the intraerythrocytic stage of the parasite asexual life cycle, and complete abrogation of gametocyte maturation^10^,^15^. These defects are not complemented by excess exogenous choline, suggesting that ethanolamine derived PC and choline derived PC are not functionally redundant^10^,^15^. These genetic data have led to the development of an *in vitro* assay to screen chemical libraries to identify inhibitors of PfPMT^9^,^10^. The antimalarial compound amodiaquine (AQ) and NSC158011, a compound identified following screening of the NCI diversity library, were found to act as non-competitive inhibitors of PfPMT and inhibit parasite development and differentiation^9^,^10^.

Structural analysis by NMR and crystallography characterized the nature and specificity of the interactions between PfPMT and substrates/inhibitors^9^,^24^,^25^,^26^,^27^. Initial residue assignment of PfPMT by NMR made it possible to characterize the interaction between PfPMT and AQ as well as it structural analog chloroquine (CQ)^9^. NMR titration studies using increasing concentrations of AQ and CQ demonstrated specific and concentration dependent binding of AQ to the enzyme and identified amino acids residues specifically altered by AQ but not CQ^9^. These studies further allowed modeling of AQ on the structure of the enzyme^9^. ^1^H, ^13^C and ^15^N chemical shifts were assigned to elucidate interactions of the enzyme with its substrate and inhibitors to enable determination of a solution structure of PfPMT^24^. Subsequent studies by Lee and colleagues helped solved the structure of the enzyme and provided detailed information about the active site and how the enzyme interacts with its substrates and product^25^. While the structural model proposed by Bobenchik and colleagues predicted a single site of binding of amodiaquine to PfPMT at the entrance of the phosphobase site^9^,^21^,^24^, structural analysis by Lee and colleagues suggested that AQ binds at two different allosteric sites^25^. One site is similar to the previously reported by Bobenchik and colleagues at the entrance of the phosphobase site and another site at the surface of the enzyme^25^.

In this study, we report the structure, inhibition and functional analysis of orthologs of PfPMT, PvPMT and PkPMT, from two human malaria parasites *P. vivax* and *P. knowlesi,* respectively. Our studies show that the structures of PMT enzymes of human malaria parasites are highly conserved and functionally active in the biosynthesis of PC from phosphoethanolamine. Interestingly, we found that *Plasmodium* PMT enzymes are targeted by the same inhibitors and our structural analysis of PvPMT with amodiaquine revealed a single binding site of the drug in a cleft distal to the active site likely involved in allosteric tuning of the enzyme. Together these findings pave the path for development of specific PMT inhibitors to target all human malaria parasites to block infection and transmission.

## Results

### Kinetic properties and inhibition of PvPMT and PkPMT

The *P. vivax* PvPMT and *P. knowlesi* PkPMT proteins are 264 amino acid residues in length and share 65% and 62% identical residues with the *P. falciparum* PfPMT (Supplemental Figure 1). The two proteins are 87.5% identical to each other and like PfPMT contain a single SAM binding domain. To characterize the biochemical properties of PvPMT and PkPMT, the kinetic parameters for phosphocholine synthesis were determined using radiometric assays at fixed concentrations of phosphoethanolamine (P-Etn) or S-adenosylmethionine (SAM)^13^. The *K_m_* values for P-Etn were 0.21 ± 0.07 mM for PvPMT and 0.10 ± 0.03 mM for PkPMT. The *K_m_* values for SAM were 0.23 ± 0.07 mM for PvPMT and 0.27 ± 0.09 mM for PkPMT (Figure 2). These values are similar to those previously reported for PfPMT^13^.

Previous studies have also shown that PfPMT activity is inhibited by the 4-aminoquinoline compounds, amodiaquine (AQ) and chloroquine (CQ) with strong inhibition obtained with AQ. The latter has been shown by NMR to alter the structure of the enzyme with ~10% of the residues in PfPMT showing a substantial change in chemical shift as a function of increasing concentrations of AQ^9^. To assess whether AQ and CQ may also inhibit the activity of PvPMT and PkPMT enzymes, transmethylation assays were performed in the absence or presence of increasing concentrations of the compounds. AQ inhibited PvPMT and PkPMT with IC_50_ values of 220 μM and 398 μM, respectively, whereas CQ inhibited the activity of PvPMT and PkPMT with IC_50_ values of 146 μM and 987 μM, respectively (Figure 3).

A chemical screen for PfPMT inhibitors using a colorometric methyltransferase assay identified the compound NSC158011 as a non-competitive inhibitor of PfPMT^10^. Studies in *P. falciparum* have shown that this compound, in the low micromolar range, inhibits intraerythrocytic development of the parasite and blocks gametocyte development^10^. Because of its suitable pharmacological properties, NSC158011 is considered a lead compound for development of more selective inhibitors of PMT enzymes. To determine whether NSC158011 can be pursued as a lead compound for inhibition of development and differentiation of different species of human malaria parasites, we examined the inhibition profile of PvPMT and PkPMT with this compound. The compound was found to inhibit PvPMT and PkPMT with IC_50_ values ~ 99 and 50 μM, respectively.

To assess the function of PvPMT and PkPMT in PC biosynthesis *in vivo*, codon-optimized cDNAs were expressed in a yeast *pem1Δpem2Δ* mutant, which lacks the two phosphatidylethanolamine methyltransfease genes *PEM1* and *PEM2*. This mutant cannot synthesize PC from PE and thus is choline auxotroph, requiring exogenous choline for survival. *pem1Δpem2Δ* expressing PvPMT or PkPMT cDNAs grew in the absence of choline, whereas strains with an empty expression vector failed to grow without choline supplementation (Figures 4A–D). As a control, addition of choline to the culture medium of *pem1Δpem2Δ* harboring an empty vector restored normal growth (Figure 4A). To determine whether the complementation of the growth defect of the *pem1Δpem2Δ* mutant by PkPMT and PvPMT was due to the synthesis of PC from ethanolamine, lipid analyses were performed on complemented and control strains to determine their steady state lipid contents. PkPMt and PvPMT expression resulted in an increase in PC content of the auxotrophic mutant from 3% to 10–15% of total phospholipids (Figure 4F). This increase is similar to that previously reported for *pem1Δpem2Δ* cells expressing the *P. falciparum* PfPMT enzyme^9^,^23^. As a control, addition of choline to the mutant cells results in an increase of PC content to 32% of total phospholipids (Figure 4E, F).

The ability of PkPMT and PvPMT to complement the loss of PC synthesis in the yeast *pem1Δpem2Δ* mutant made it possible to examine the effect of AQ and NSC158011 on the enzymes and PC metabolism *in vivo*. Growth of *pem1Δpem2Δ*+PkPMT and *pem1Δpem2Δ*+PvPMT strains was significantly inhibited following treatment with AQ and NSC158011 (Figures 5). Consistent with a direct effect on PMT activity, AQ and NSC158011 had no effect on the growth of these strains in media supplemented with choline (Figure 5). Lipid analyses revealed that the growth inhibition by AQ and NSC158011 correlates with a specific decrease in PC biosynthesis. At 200 μM AQ reduced the PMT-dependent cellular PC content by 50 to 65%, and at 20 μM NSC158011 inhibited PC biosynthesis by ~20 and 26% for PkPMT and PvPMT, respectively. At these concentrations these compounds had little to no effect on the synthesis of PE (Figure 6A and C). Consistent with previous findings^9^, the inhibition of PC synthesis by AQ or NSC158011 was accompanied by an increase in PI content, resulting in a PC/(PI + PA) ratio reduction by 4 to 5-fold for AQ and ~2-fold for NSC158011 (Figure 6B and D).

### X-ray structure of PvPMT and PkPMT

PkPMT, PvPMT and PvPMT-AQ structures were solved with the diffraction amplitudes extending to 1.9, 1.4 and 1.5 Å, respectively (Figures 7, 8, 9). The resolution of these structures allowed determination of proximity of hydrogen bond donors and acceptors, enabling detailed understanding of the interaction of the protein residues with substrates and inhibitors. The general structural folds of PvPMT and PkPMT are very similar to that previously reported for PfPMT^26^ with all atom RMSD = 0.452 Å (Figure 10). Comparison of the three structures show that apart from local changes attributable to differences in protein sequences, the secondary structure elements are constant. We were unambiguously able to build most of the structure of PvPMT into the obtained electron density maps, except for a few residues at the N-terminus. The SAM methyl donor is bound between the Rossmann fold motif containing a highly conserved glycine-rich sequence (Figure 7A, B). SAM forms hydrogen bonding and hydrophobic interactions with several of PvPMT residues and water molecules (Figure 7C). Hydrogen bonds couple the N6 and N1 of the adenine ring of SAM to the carboxyl group of D107 and the backbone amino group of I108. The hydroxyl groups of ribose O2 and O3 are hydrogen bonded to the side chain of D82 and a water molecule. The carboxyl group of SAM is hydrogen bonded to the backbone amino group/nitrogen of I33. In addition, the backbone amide is hydrogen bonded to the carbonyl oxygen of G60 and two water molecules (Figure 7C). The S-methyl group of SAM is within 3.2 Å and 4.8 Å distance from the putative catalytic dyad, consisting of Y16 and H129, respectively. Van der Waals interactions are observed in the SAM binding site with several isoleucine residues. Phosphate anion is hydrogen bonded to the hydroxyl groups of Y24, Y157 and Y178 and the guanidino group of R176 (Figure 7C).

The general structure of PkPMT is almost identical to that of PvPMT and PfPMT with an all atom alignment RMSD of 0.80 Å (Figure 8A and Figure 10). The first 20 residues of PkPMT were disordered, but clear electron density was present for residues 21 to 265 and SAM (Figure 8B). Interactions of SAM with the active site residues and water are similar to the ones seen in PvPMT structure. The methionine S-methyl group of SAM is at 5.1 Å from the putative catalytic H130 (Figure 8C).

The 1.5Å resolution structure of PvPMT bound to AQ was similar to that of PvPMT alone (all atom alignment RMSD = 0.18 Å) (Fig. 9 and Supplemental Figure 2). The structure of PvPMT-AQ shows that the drug binds to PvPMT at a unique location adjacent to the active site (Figure 9). This is consistent with previous studies by Bobenchik and colleagues^9^ that indicated the presence of a single binding site for AQ on PfPMT and suggests that the reported structure for AQ binding on a second site on PfPMT by Lee and colleagues^25^ is likely to represent a crystallographic artifact. A close look at the unit cell parameters (Table 2) reveals that unit axis do change as a result of soaking crystals with AQ making isomorphous replacement of the apo structure impossible into the crystal lattice of inhibitor soaked lattice. The 2° in the beta axis results in a translation of ~2Å of chain B along that axis (Figure 9C and Supplementary Figures 3, 4 and 5). Furthermore, the buried surface area between the symmetry related molecules that trap AQ increase from 326 Å^2^ to 524 Å^2^ as a result of inhibitor binding. For AQ binding, a hydrophobic pocket is made up from the side chains of I39, L210 and L213 that seem to facilitate the van der Waals interactions necessary for the binding of the bulk of the phenolic side of the molecule (Fig. 9C). In addition to the hydrogen bonding interaction from the carboxylate oxygen of E214, there is also a potential hydrogen bonding/electrostatic partner to the phenyl oxygen from a main chain carbonyl of G36 (Fig. 9C). AQ makes multiple interactions with its protein-binding partner as well as with residues from the crystallographic symmetry related molecule (Figure 9C) (residues from the symmetry related molecule are indicated with an asterisk). The bridging amino group (N2) of AQ forms a hydrogen bond with a water molecule while the chlorine group interacts with *E185 of the symmetry related molecule. The chloroquinoline ring of AQ π-stacks with *Y132 of the symmetry related molecule. The carboxylate group of *E185 is 0.3Å closer for a tighter interaction with the Cl moiety of the inhibitor (Figure 9C). Several van der Walls and hydrophobic interactions help AQ bind to the outer surface of the protein (Figure 9C).

## Discussion

In this study we have examined the biochemical properties, structures and inhibition of the PMT orthologs from *P. vivax* and *P. knowlesi* and compared their structures to that of *P. falciparum* PMT. Our data indicate that PMT enzymes from different human malaria parasites have similar biochemical properties, structures and inhibition profiles and thus are valid targets for development of antimalarials selective against different parasite species.

Our biochemical analyses revealed that *K*_m_ values for the substrate P-Etn and cofactor SAM are similar for all the three malarial PMT enzymes (Pv, Pk and Pf). Though sequence identity at the amino acid level when compared with PfPMT is 64% (PvPMT) and 62% (PkPMT), residues of SAM binding domain and phosphobase are highly conserved^28^. PMT activity has been previously shown to follow a random sequential Bi Bi kinetic mechanism, indicative of 2 substrate 2 product reaction wherein binding of either of the substrate only modestly affect binding of the other^29^,^30^. Inhibition studies showed that the 4-aminoquinoline compounds, AQ and CQ, and the NCI compound NSC158011 previously found to inhibit PfPMT also inhibit PvPMT and PkPMT enzyme catalysis. Similar to PfPMT, the activity of NSC158011 was stronger compared to that of AQ and CQ. Although the sensitivity of the three enzymes to these compounds can vary from 2 to 10-fold, the amino acid sequence of the AQ binding site predicted by Bobenchik and colleagues and the site determined by the PvPMT-AQ crystal structure is mostly conserved between all three enzymes with the exception of E209 (in PvPMT and PkPMT) residue, which is involved in a weak hydrogen bond with the chloroquinoline ring of AQ and corresponds to Q212 in PfPMT. Based on previously published crystallographic evidence, the structure of PfPMT contains at least two binding sites for the amodiaquine inhibitor. The first site blocks the entrance/exit channel of the p-Etn/p-Cho substrate/product, and the second site is distal to the active site in a cleft between helices α5 and α9. Although the two binding sites are almost identical in protein sequence between PfPMT and PvPMT we were crystallographically able to demonstrate the binding only in the second site. The second binding site serves a likely allosteric role that brings about minute structural changes via second and third shell hydrogen bonding interactions that affect catalysis. We hypothesize these motions may not be large enough to be trapped by a static X-ray structure (Supplementary Figure 2).

At this stage of the analysis, it remains unknown what additional residues in the sequences of PMT enzymes might be important for the interaction of the drugs with their binding site. Nevertheless, the residues in this novel allosteric site are conserved enough for a single inhibitor to inhibit all three enzymes.

Crystal structures of PvPMT and PkPMT bound to the substrate SAM and phosphate demonstrates that the PMT active site is hydrophobic. The general structural folds of PvPMT, PfPMT and PkPMT are very similar with all atom RMSD = 0.452 Å (Figure 8). The active site residues are well conserved amongst enzymes from *P. falciparum*, *P. vivax* and *P. knowlesi*. The accommodative nature of the active site shown by the structural comparisons of PfPMT in complex with pEA, AdoMet (substrates), pCho (product) and AdoCys-pEA (a dead-end complex) suggests the plausibility of further optimization of an inhibitor to its highest potency. An active site inhibitor could potentially inhibit all three (Pf, Pv, Pk) PMTs and such an inhibitor can be developed as a broad-spectrum antimalarial drug.

Inhibition studies confirmed that drugs known to inhibit PfPMT also inhibit PvPMT and PkPMT. Although several lines of evidence suggest that AQ and CQ exert their antimalarial activity primarily in the parasite's digestive vacuole by inhibiting heme polymerization, analyses of genetic crosses as well as biochemical studies have indicated that other mechanisms of action might also exist and might account for their activity and the complex resistance profiles seen with these drugs^31^. Similarly, NSC158011 identified following screening of the NCI Diversity chemical library, inhibited all three PMT enzymes from human malaria parasites. This compound was also found to inhibit gametocyte development consistent with the inhibition of PfPMT's important function in sexual differentiation. AQ and NSC158011 are allosteric inhibitors of malarial PMTs exhibiting noncompetitive enzyme inhibition^10^. Crystal structure of AQ in complex with PvPMT confirmed previous NMR data, which identified the binding site of AQ by NMR chemical shift experiments^9^.

Our characterization of PMT enzymes from different human malaria parasites has identified several lead inhibitors representing at least two chemical classes^9^,^10^. Further studies to optimize these lead compounds using available structural data will help enhance the selectivity of the inhibitors towards this family of methyltransferases versus other common methyltransferases to reduce their toxicity.

## Methods

### Purification, crystallization and data collection of PkPMT and PvPMT

The genes encoding PkPMT and PvPMT were expressed from pET-28a vector with an N-terminal 6-His fusion tag in *E.coli* strain BL21 (DE3) in LB medium supplemented with 50 μg/mL of kanamycin. For large scale expression, 2 L culture was incubated for two days at 30° C with shaking at 200 rpm. The cells were harvested via centrifugation and, suspended in 50 mM Tris-HCl buffer (pH 7.5) containing 5 mM imidazole, and 0.5 M NaCl. The cells were lysed by sonication on ice using a Fisher Scientific 550 Sonic Dismembrator (Fisher). The proteins were purified using a Nickel HisTrap column followed by cleavage of the His-tag with thrombin (GE Healthcare). Removal of the His-tag was verified by SDS-PAGE. Prior to crystallization, the cleavage product was further purified via gel filtration chromatography using a HiLoad 16/60 Superdex 75PG column. For storage, the enzymes were dialyzed into a solution consisting of 100 mM NaCl buffered with 20 mM Tris-HCl (pH 7.5). PkPMT was crystallized using the hanging drop vapor diffusion method at 9° C. The protein solution was at a concentration of 15 mg/mL containing 20 mM Tris-HCl (pH 7.5), 100 mM NaCl, 2 mM EDTA, 5 mM β-mercaptoethanol (β-ME), and 5 mM S-adenosylmethionine (SAM). Mother liquor consisted of 100 mM Na-acetate (pH 5.0), and 20% w/v PEG 3,350. Prior to data collection, PkPMT crystals were transferred into a cryoprotectant solution containing 10% glycerol, followed by vitrification in liquid nitrogen (LN_2_). Diffraction images were recorded at the Advanced Photon Source (APS) beamline ID-21-G (Argonne National Laboratory, Lemont, IL) using a Rayonix MX-300 detector. The crystals diffracted to 1.9Å resolution and were consistent with the space group P2_1_2_1_2. PvPMT was crystallized via sitting drop vapor diffusion method at 4° C. The protein concentration was 15 mg/mL and contained 100 mM NaCl, 10 mM EDTA, 10 mM β-ME, and 5 mM SAM buffered with 20 mM Tris-HCl (pH 7.5). Mother liquor consisted of 200 mM NH_4_-formate and 20 % w/v PEG 3,350. Prior to data collection, PvPMT crystals were transferred to a cryoprotectant solution containing 30% w/v PEG 3,350 followed by immediate vitrification in LN_2_. In order to obtain an AQ bound crystal structure of PvPMT, a saturated solution of AQ in DMSO was added directly to the drop containing PvPMT crystals at 5% of the total drop volume and incubated overnight. Prior to data collection, PvPMT-AQ crystals were transferred to a cryoprotectant solution containing 30% w/v of PEG 3,350 followed by vitrification by immersion in LN_2_. The diffraction data for the apo and ligand bound PvPMT crystals were recorded at APS beamline ID-21-F using a Rayonix MX-225 detector. The crystals were consistent with the space group P2_1_ and diffracted to 1.4 and 1.5 Å, respectively. AQ is identified as CQA in the PDB code 4MWZ.

### Structure solution and refinement of PkPMT and PvPMT

The diffraction images were indexed and integrated using the XDS package^32^. The structures of PkPMT and PvPMT were solved with molecular replacement with PHASER^33^, using the molecular coordinates of the phoshpoethanolamine methyl transferase from *Plasmodium falciparum* (PDB code 3UJ6). Partial structure solution from PHASER was then subjected to iterative cycles of automated and manual model building with BUCCANEER^34^ and COOT^34^. Solvent atoms were placed with PHENIX^35^ and validated in COOT. Final structure refinement was performed with REFINE in PHENIX. Data collection and structure refinement statistics can be found in Table 1.

### Enzyme Kinetics

For enzyme kinetics studies recombinant histidine-tagged proteins were expressed using 1 mM IPTG for 4 h, purified using Ni-affinity purification and dialyzed. As expected, purified proteins migrated in SDS-PAGE gels at approximately 31 kDa. The purified proteins were stored in HEPES-KOH buffer (pH 7.4) at 4°C and were assayed within a month of purification. During this time, the integrity of the protein was assessed by SDS-PAGE. The kinetic properties of enzymes were assessed using radiometric assay wherein incorporation of radioactive labeled methyl donor (100nCi [methyl-^14^C] SAM, Perkin elmer NEC363010UC) or (200nCi [methyl-^3^H] SAM, Perkin Elmer NET155H250UC) was measured^13^. The enzymatic assay conditions were carried out in 100 μl volume containing 0.1 M Hepes, KOH (pH8.6), 2 mM Na_2_EDTA and10% glycerol. For NSC158011 related studies, 10% DMSO was added to the reaction mixture. Purified enzyme (322 nM) was used in each reaction and the reactions were incubated at 37°C for 30 minutes, thereafter the reaction was terminated using 1 ml cold water. Afterwards, the product was purified by batch purification using AG (H+ resin) (Biorad # 142-1451) using the protocol provided by the manufacturer. The product was eluted from resin using 1.5 ml of 0.1 N HCl and total radioactivity was measured using the scintillation cocktail (Biosafe II).

To determine the *K*_m_ value for P-Eth, enzymatic reactions were carried out with 0-0.8 mM of P-Eth at constant 1.5 mM SAM. Similarly *K*_m_ value for SAM was carried with 0–0.6 mM SAM at constant 2 mM P-Ethanolamine (P-Etn). The inhibition of PvPMT and PkPMT by AQ, CQ and NSC158011were also examined using the same radiometric PMT transmethylation assay. Enzyme activity was evaluated at 0, 100, 500 and 1000 μM of the inhibitor at a fixed concentration of P-Eth (2 mM) and SAM (1.5 mM). To calculate IC_50_ the data was fitted to non-linear regression analysis in the following equation Y = 100/(1 + ([X]/IC_50_)), where Y is % enzyme activity and X is inhibitor concentration using Graph pad software.

### Statistical analyses

Statistical analyses were performed using an unpaired Student's t-test. Differences were considered statistically significant when *P* < 0.05.

### Yeast Growth

*pem1Δpem2Δ-pYES2.1, pem1Δpem2Δ-pYES2.1-PkPMT, pem1Δpem2Δ-pYES2.1-PvPMT, and pem1Δpem2Δ-pYES2.1-PfPMT* yeast strains were pre-grown overnight in uracil dropout synthetic galactose (4%) (SG-URA) medium supplemented with 2 mM ethanolamine (Etn). Cells were diluted to an A_600_ = 0.001 in fresh SG-URA medium supplemented with 2 mM Etn in the presence or absence of 100 μM Choline (Cho). Cells were grown at 30°C and the growth was monitored by measuring the A_600_. For the growth inhibition study, *pem1Δpem2Δ-pYES2.1-PkPMT, pem1Δpem2Δ-pYES2.1-PvPMT, and* BY4741*-pYes2.1 (wild-type)* strains were pre-grown in SG-URA medium supplemented with 200 μM Etn and diluted to an A_600_ = 0.001 in fresh SG-URA medium supplemented with 2 mM Etn in the presence or absence of 100 μM Cho, 100 μM AQ, and/or 10 μM NSC158011. At least three independent experiments were performed for each assay.

### Phospholipid analyses

BY4741*-pYes2.1 (wild-type)* strains and the *pem1Δpem2Δ strains* harboring *pYES2.1* vector, *pYES2.1-PkPMT, pYES2.1-PvPMT* or *pYES2.1-PfPMT* were pre-grown to mid-log phase at 30°C in SG-URA medium supplemented with Etn (2 mM) and Cho (2 mM). The cells were harvested by centrifugation, washed twice with dH_2_O, and diluted to A_600_ = 0.02 (with an exception of *pem1Δpem2Δ-pYES2.1* which was diluted to A_600_ = 0.025) in 50 mls of SG-URA medium supplemented with Etn (2 mM) and grown to A_600_ ~ 1.5. The cells were harvested by centrifugation, washed twice with dH_2_O. The lipids were extracted as described previously^36^,^37^. The phospholipids were separated by thin layer chromatography (TLC) on Silica 60 plates TLC plates in the solvent system of chloroform, methanol, 2-propanol, 0.25% KCl, triethylamine (30:9:25:6:18, vol/vol). The resolved lipids were visualized by spraying with 0.1% aqueous 8-anilino-l-naphthalene sulfonic acid (ANSA) and exposure to ultraviolet light. Each lipid class was excised from the plate and quantified by measuring phosphorus^38^. The results are shown as the percentage of total lipid phosphorus in each phospholipid fraction. Data are means ± S.E. for three independent experiments. For lipid analysis of yeast strains treated with AQ or NSC158011, the yeast strains were pre-grown to mid-log phase at 30°C in SG-URA medium supplemented with 2 mM Etn, diluted to A_600_ = 0.025 in 50 mls of SG-URA medium supplemented with Etn (2 mM) and AQ (200 μM) or NSC158011 (10 μM) grown to A_600_ = 1.5. After the growth, lipids were extracted and analyzed as described above. Phospholipid Analysis following treatment with NSC158011 was as follows: *pem1Δpem2Δ* strains with pYES2.1-PkPMT or pYES2.1-PvPMT vector were grown in synthetic uracil dropout medium with 4% galactose as a carbon source (SG-Ura) supplemented with 2 mM Ethanoamine. Cells in mid-log phase were harvested by centrifugation and washed twice by cold water and recentrifugation. The cells were suspended in SG medium, at an A_600_ of 0.03 in a volume of 2 ml in the absence or presence of 20 uM NSC158011. Radiolabeling was initiated by adding 8 μCi of [^33^P] orthophosphoric acid, and growth was continued at 30°C for 17 hour (for control strains) to 22 hours (NSC treated strains). Labeled phospholipids were extracted as previously described^39^ and the lipid classes were resolved by thin layer chromatography on silica-60 TLC plates in the solvent system of chloroform, methanol, 2-propanol, 0.25% KCl, triethylamine (30:9:25:6:18, v/v). The resolved radioactive lipids were visualized by Phosphorimager (GE Typhoon) and quantified by liquid scintillation spectrometry as described^37^.

## Author Contributions

**Figure 1:**
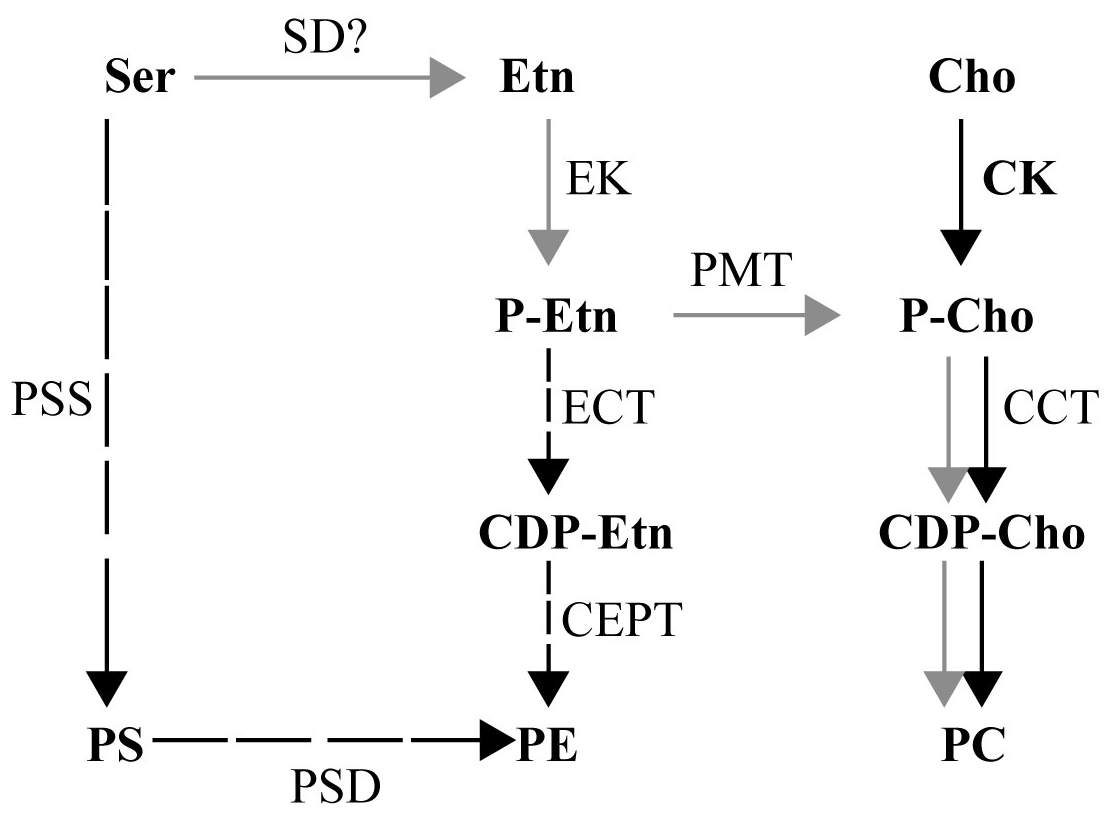
Metabolic pathw ays for synthesis of PC in *P. falciparum*. PC, phosphatidylcholine; PE, phosphatidylethanolamine; PS: phosphatidylserine. The CDP-choline pathway is shown is black arrows and the SDPM pathway is gray arrows. Cho, choline; CDP, cytidine diphosphate; CDP-cho, CDP-choline; CCT: CTP:phosphocholine cytidylyltransferase; ECT: CTP:phosphoethanolamine cytidylyltransferase;Ser: serine; Etn: ethanolamine; CDP-Etn, CDP-ethanolamine; EK: ethanolamine kinase; CK: choline kinase; PSS: PS synthase; PSD: phosphatidylserine decarboxylase; ECT; CEPT: CDP-choline/ethanolamine: 1,2 diacylglycerol choline/ethanolamine phosphotransferase; SD: serine decarboxylase (SD gene is unknown).

**Figure 2:**
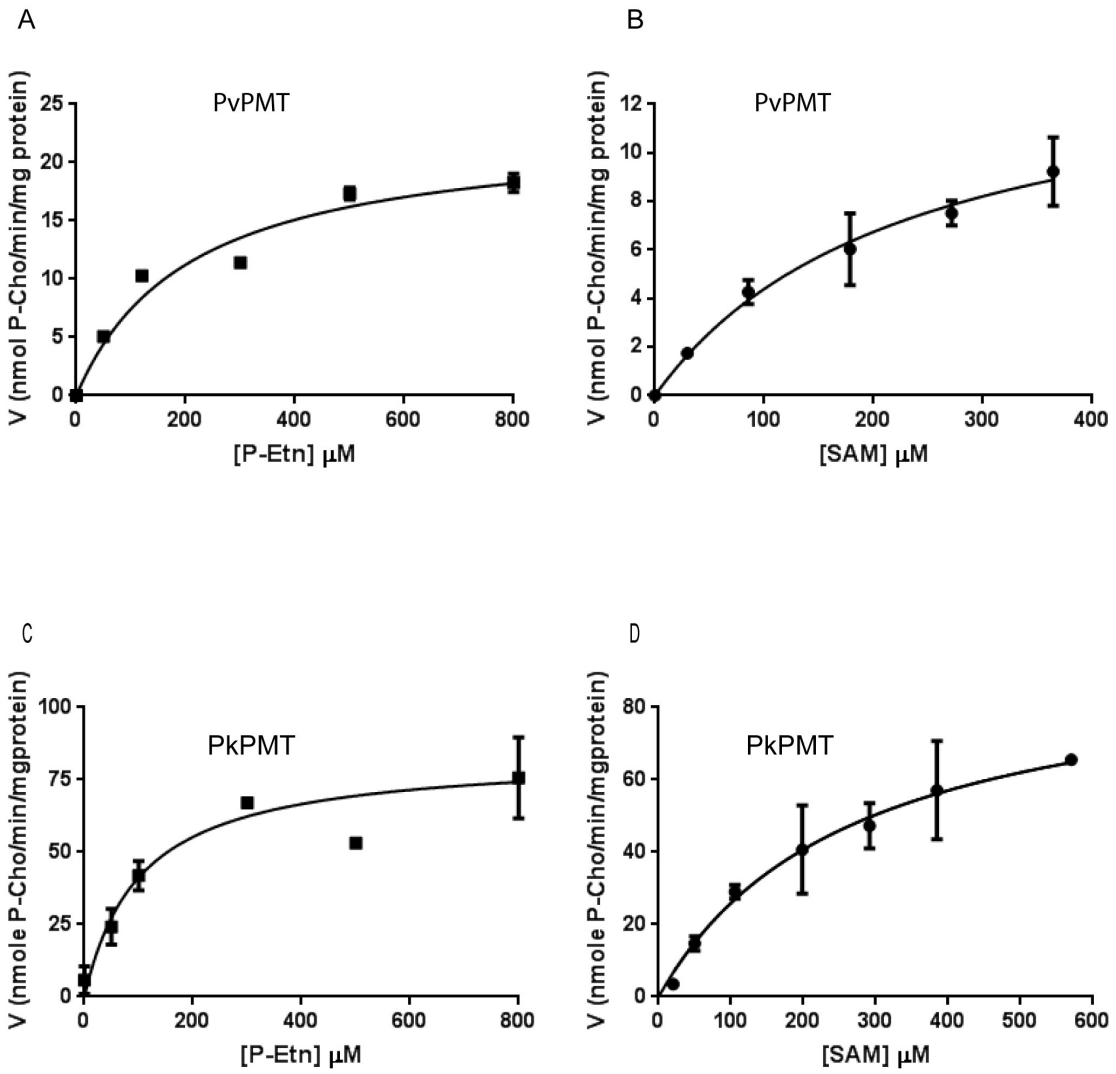
Analysis of kinetic parameters of *P.vivax* (top panel) and *P. knowlesi* PMT enzymes (bottom panel). The *K_m_* values for phosphoethanolamine (P-Etn) was determined under standard assay conditions with varying concentrations of P-Eth (0–800 μM) and at constant S-adenosylmethionine (SAM) concentration of 1.5 mM. The *K_m_* values for SAM were determined under standard assay conditions with varying concentrations of SAM (0–600 μM) and at constant P-Etn concentration of 2.0 mM. All data were fitted to the Michaelis-Menten equation, *v* = (*k_cat_* [Et][S])/(*K_m_* + [S]) using Graph pad software.

**Figure 3:**
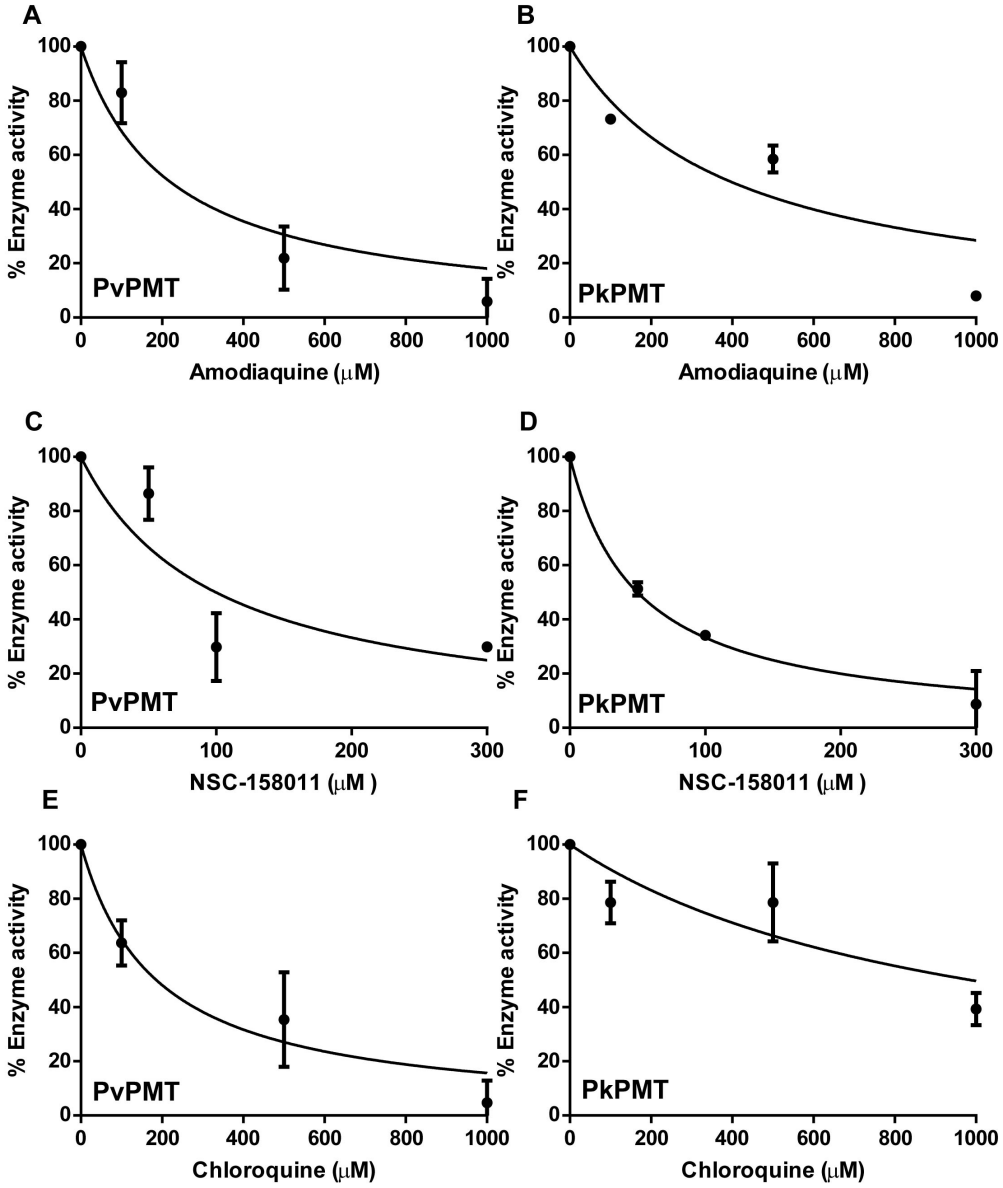
Amodiaquine (AQ) and NSC158011 inhibit the activity of PvPMT and PkPMT. Enzyme inhibition assays with different concentrations of Amodiaquine (AQ), NSC158011 and Chloroquine (CQ) were carried out at constant P-Etn (2 mM) and SAM (1.5 mM) concentrations. The activity in the absence of any drug was taken as 100% and % enzyme activity was calculated in the presence of different drug concentrations (100, 500, 1000 μM). To determine the concentration that results in 50% inhibition of enzyme activity (IC_50_), the data were fitted to a non-linear regression analysis in the following equation Y = 100/(1 + ([X]/IC50)), where Y is % enzyme activity and X is drug concentration using Graph pad software.

**Figure 4:**
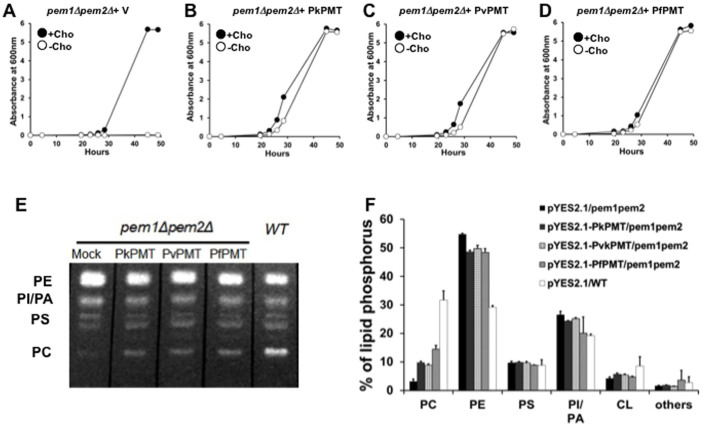
PkPMT and PvPMT complement the loss of PC synthesis from ethanolamine in yeast. A-D: represent growth curves of *pem1Δpem2Δ-pYes2.1* (A) and *pem1Δpem2Δ-pYES2.1-PkPMT* (B), *pem1Δpem2Δ-pYES2.1-PvPMT* (C), or *pem1Δpem2Δ-pYES2.1-PfPMT* (D) strains cultured in minimal medium containing 4% galactose and 2 mM ethanolamine in the presence or absence of 100 μM choline (Cho). E and F: Analysis of the phospholipid content of *pem1Δpem2Δ-pYes2.1, pem1Δpem2Δ-pYes2.1*-*PkPMT*, *pem1Δpem2Δ-pYes2.1*-*PvPMT* and *wild type-pYes2.1* strains. The yeast strains were grown in minimal medium containing 4% galactose and 2 mM ethanolamine. The lipids were extracted, separated by TLC and visualized under UV light after ANSA spray (E). Each lipid class was scrapped off from the TLC plate and quantified by measuring phosphorous (F) as described in experimental procedures. The graph is the percentage of total lipid phosphorous in each lipid fraction. The data are represented as the means ± S. E. of three independent experiments.

**Figure 5:**
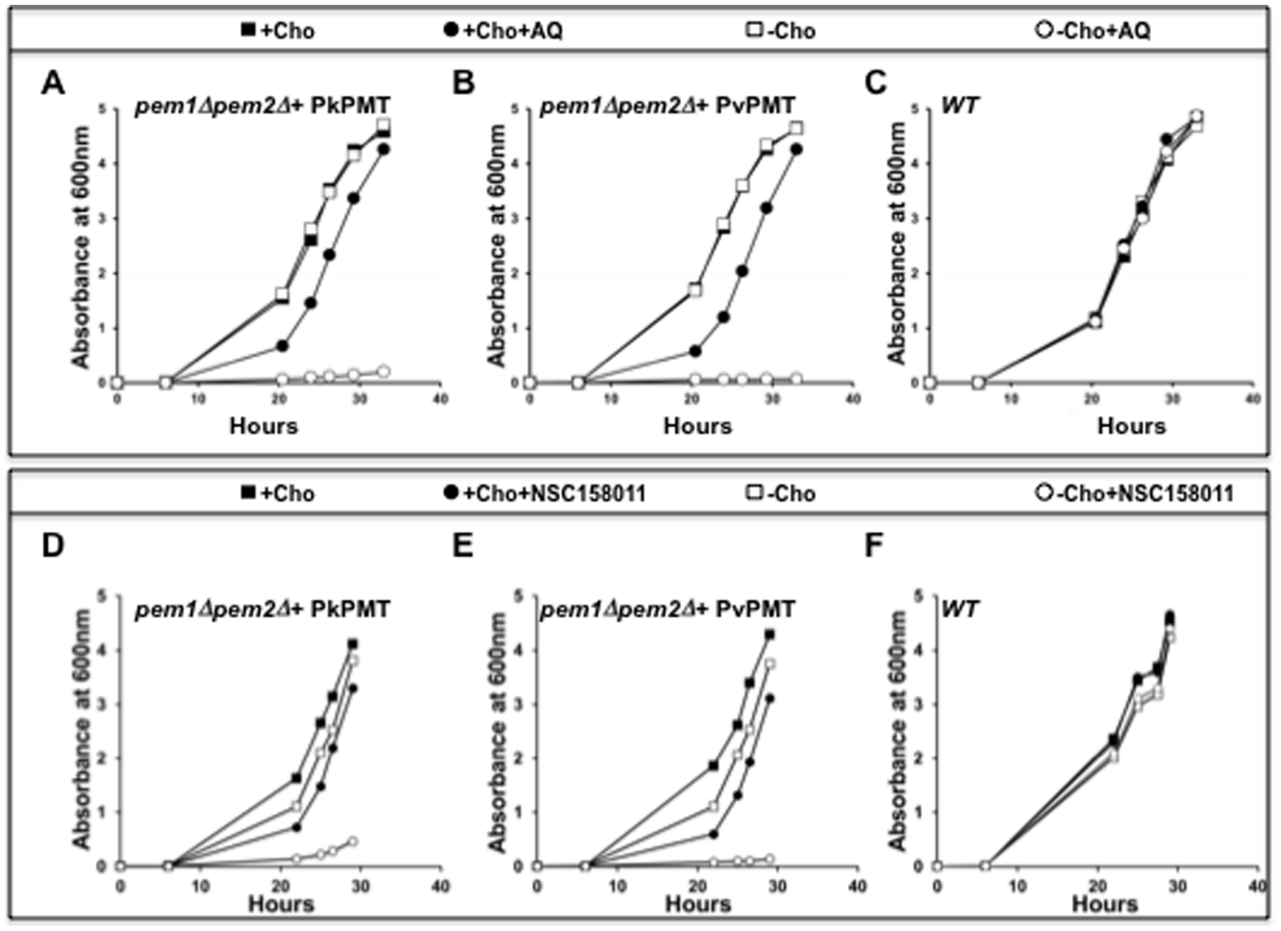
PMT-dependent inhibition of growth and PC biosynthesis by amodiaquine and NSC158011. (A–C): Growth curves of *pem1Δpem2Δ-pYES2.1-*PkPMT (A), *pem1Δpem2Δ-pYES2.1-*PvPMT (B), or *Wild Type-pYES2.1-* (C) strains grown in minimal medium containing 4% galactose and 1 mM ethanolamine in the presence or absence of 100 μM choline and 100 μM amodiaquine (AQ) as indicated. Growth curves of *pem1Δpem2Δ-pYES2.1-PkPMT* (D), *pem1Δpem2Δ-pYES2.1-*PvPMT (E), or *Wild Type-pYES2.1-* (F) strains maintained in minimal medium containing 4% galactose and 1 mM ethanolamine in the presence or absence of 100 μM choline and 10 μM NSC158011.

**Figure 6:**
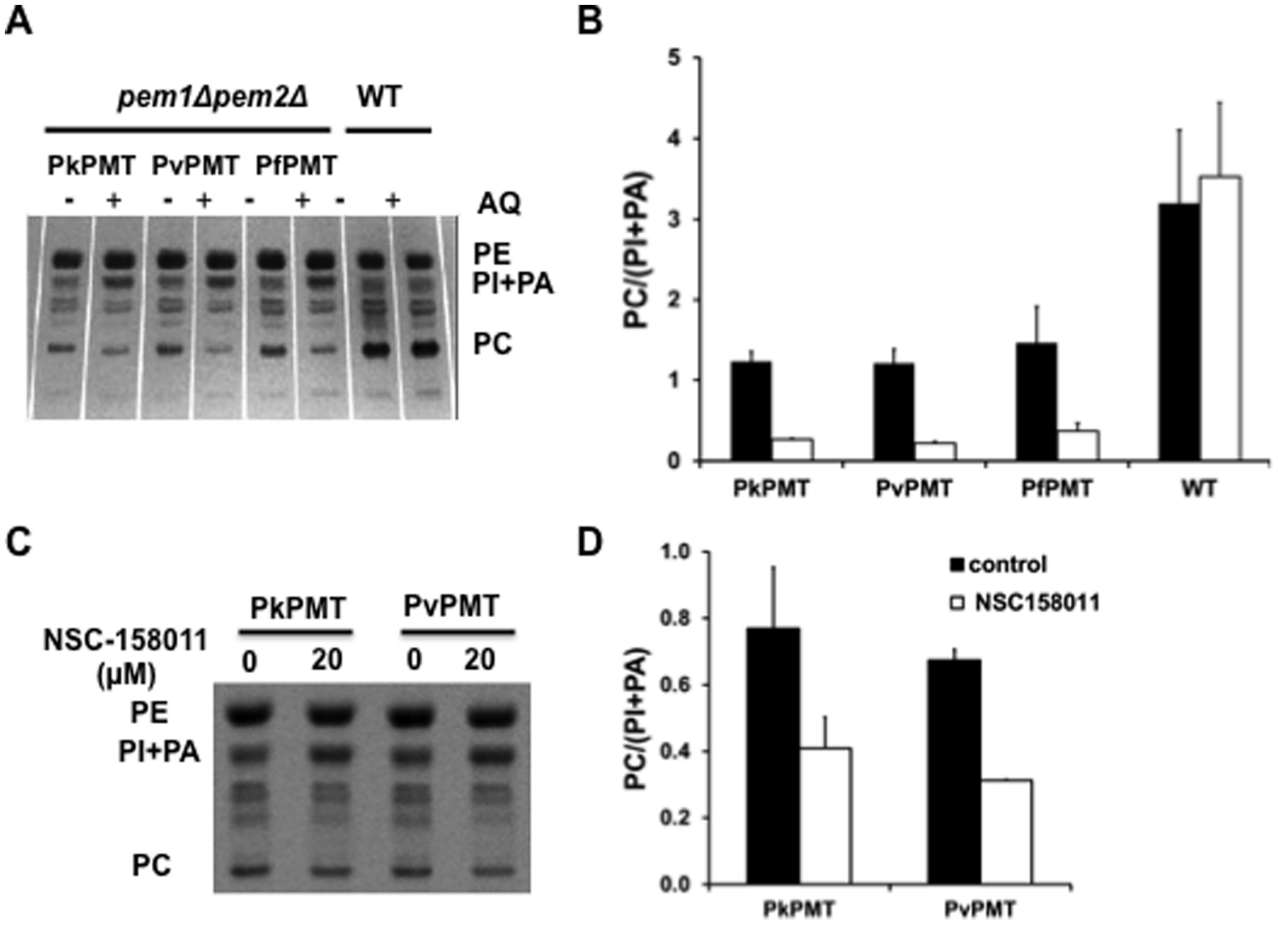
PMT-dependent inhibition of PC biosynthesis by AQ and NSC158011. To assess the effect of AQ on phospholipid metabolism, *pem1Δpem2Δ-pYES2.1-PkPMT*, *pem1Δpem2Δ-pYES2.1-PkPMT* and wild-type strains were grown in minimal medium containing 4% galactose and 2 mM ethanolamine in the absence or presence of 200 μM AQ, lipids were extracted and separated by TLC (A). Each lipid class was scrapped off the TLC plate and quantified by measuring phosphorus (B). The effect of NSC158011 on phospholipid biosynthesis was determined by [33P] orthophosphoric acid labeling as described in Material and Methods. Lipids were separated by TLC (C) and quantified by liquid scintillation spectrometry (D). For all assays, data were used to calculate the PC/PI + PA ratio. The data are represented as the mean ± S.D. of at least two independent experiments.

**Figure 7:**
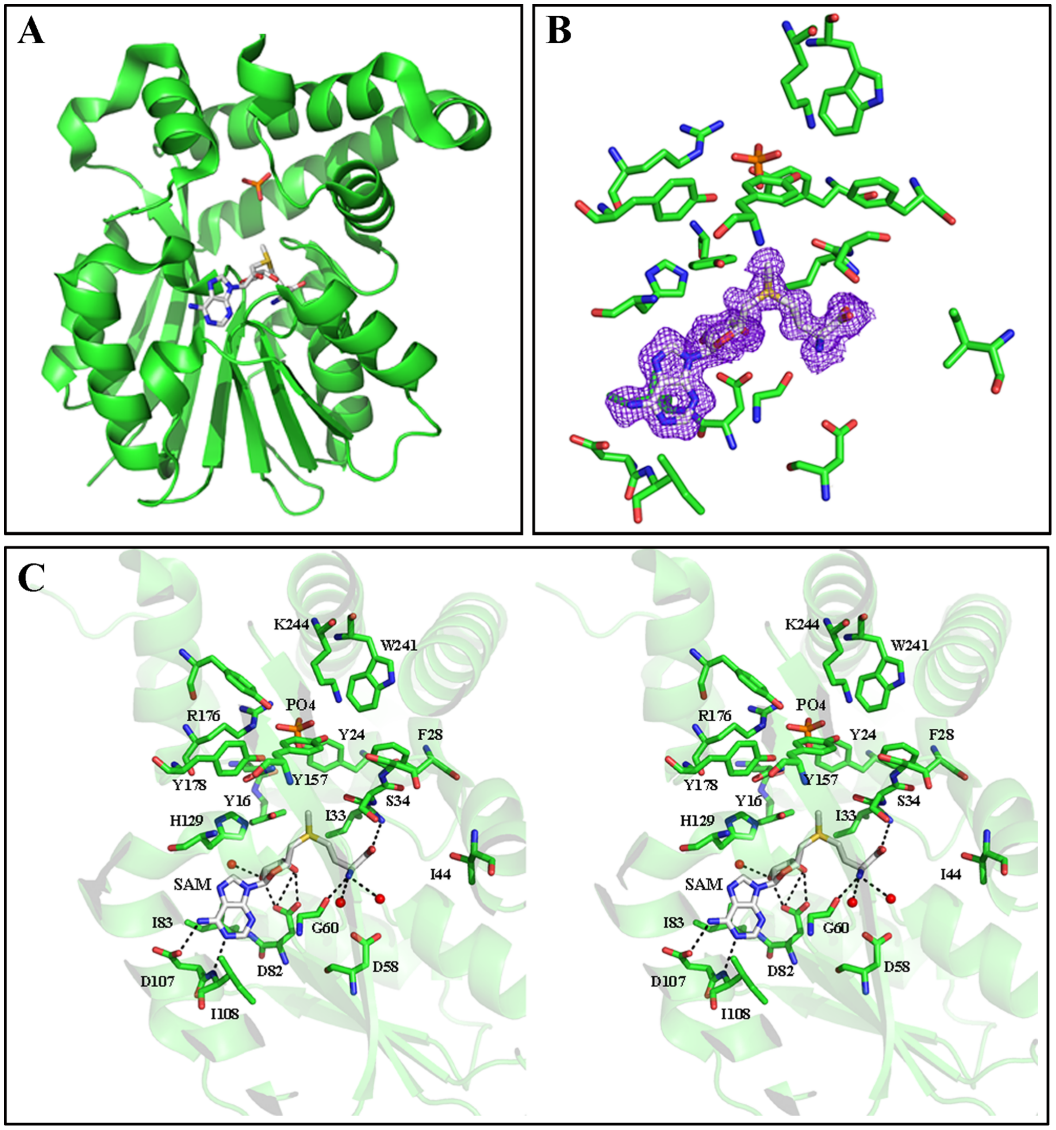
An overview of the crystal structure of PvPMT in complex with SAM and PO4 (PDB code: 4IV0). (A) Folds of PvPMT shown as cartoon (green) with SAM (gray) and PO4 (red) in stick representation. (B) Omit *σ_A_*-weighted *mF*_o_-*F*_c_ electron density map for SAM bound to PvPMT active site (contour level at 1.0 σ). (C) Stereo view of active site residues of PvPMT (green) interacting with SAM (gray) and PO4 (red). Water is represented as spheres (red). Hydrogen bonds (<3.5 Å) are indicated as dashed lines.

**Figure 8:**
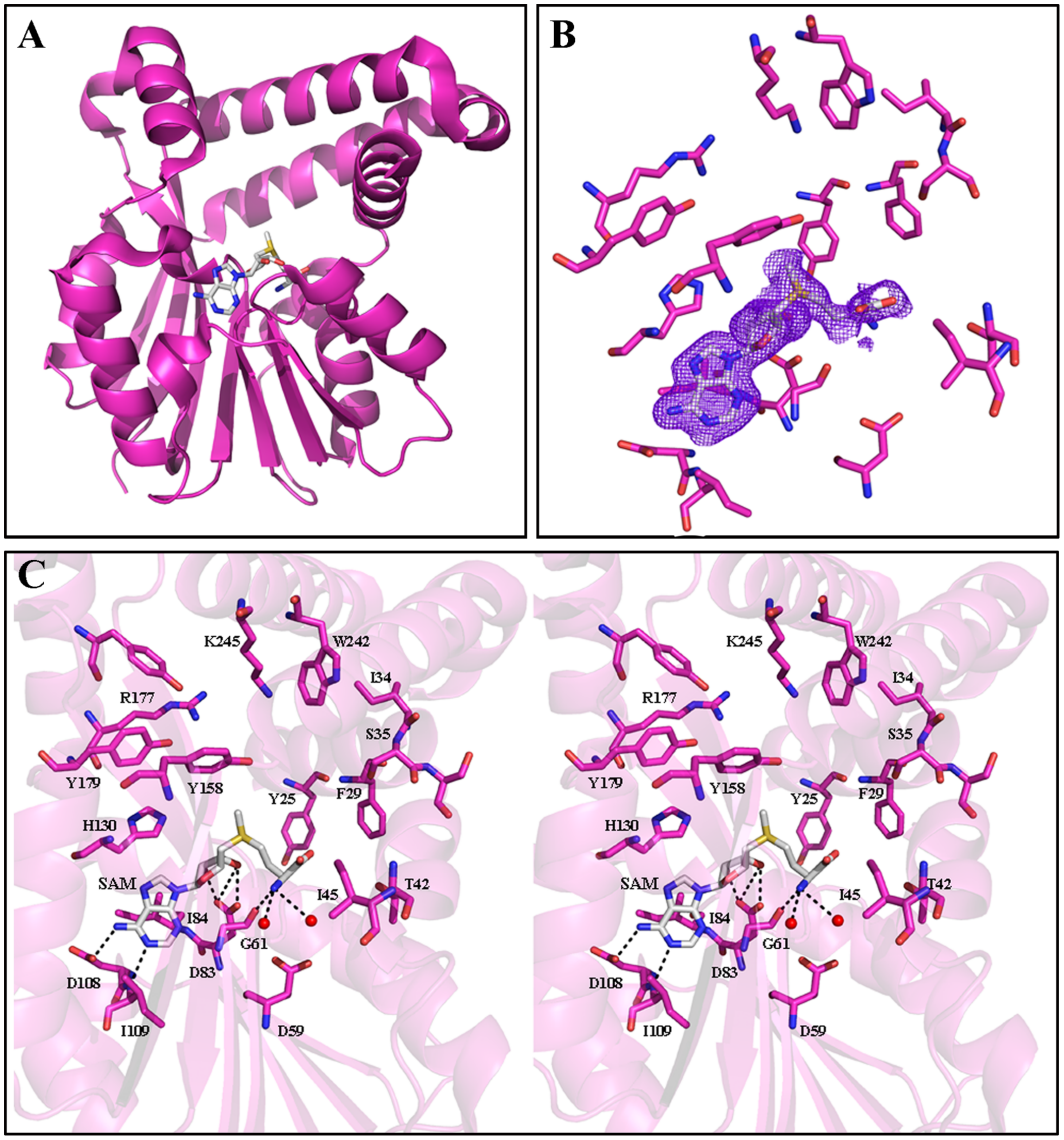
An overview of the crystal structure of PkPMT in complex with SAM (PDB code: 4IV8). (A) Folds of PkPMT shown as cartoon (pink) with SAM (gray) in stick representation. (B) Omit *σ_A_*-weighted *mF*_o_-*F*_c_ electron density map for SAM bound to PkPMT active site (contour level at 1.0 σ). (C) Stereo view of active site residues of PkPMT (pink) interacting with SAM (gray). Water is represented as spheres (red). Hydrogen bonds (<3.5 Å) are indicated as dashed lines.

**Figure 9:**
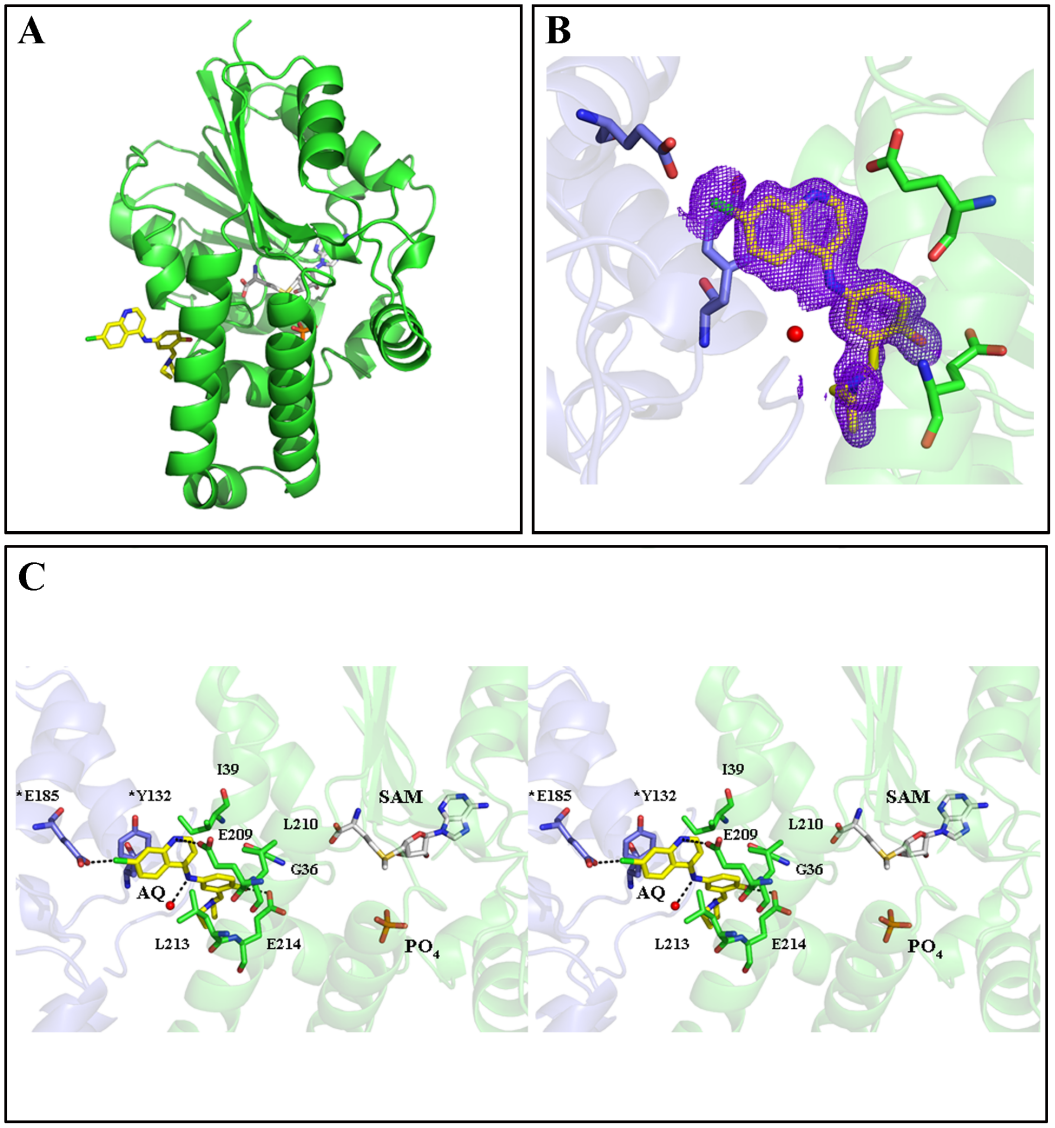
An overview of the crystal structure of PvPMT in complex with SAM, PO4 and amodiaquine (PDB code: 4MWZ). (A) Folds of PvPMT shown as cartoon (green) with SAM (gray) and AQ (yellow) in stick representation. (B) Omit *σ_A_*-weighted *mF*_o_-*F*_c_ electron density map for AQ bound to PvPMT (contour level at 1.0 σ). (C) Stereo view of residues of PvPMT (green) interacting with AQ (yellow), SAM (gray) and PO4 (red). Symmetry molecule is shown in pale blue while residues from the symmetry molecule are indicated with *. Water is represented as spheres (red). Hydrogen bonds (<3.5 Å) are indicated as dashed lines.

**Figure 10:**
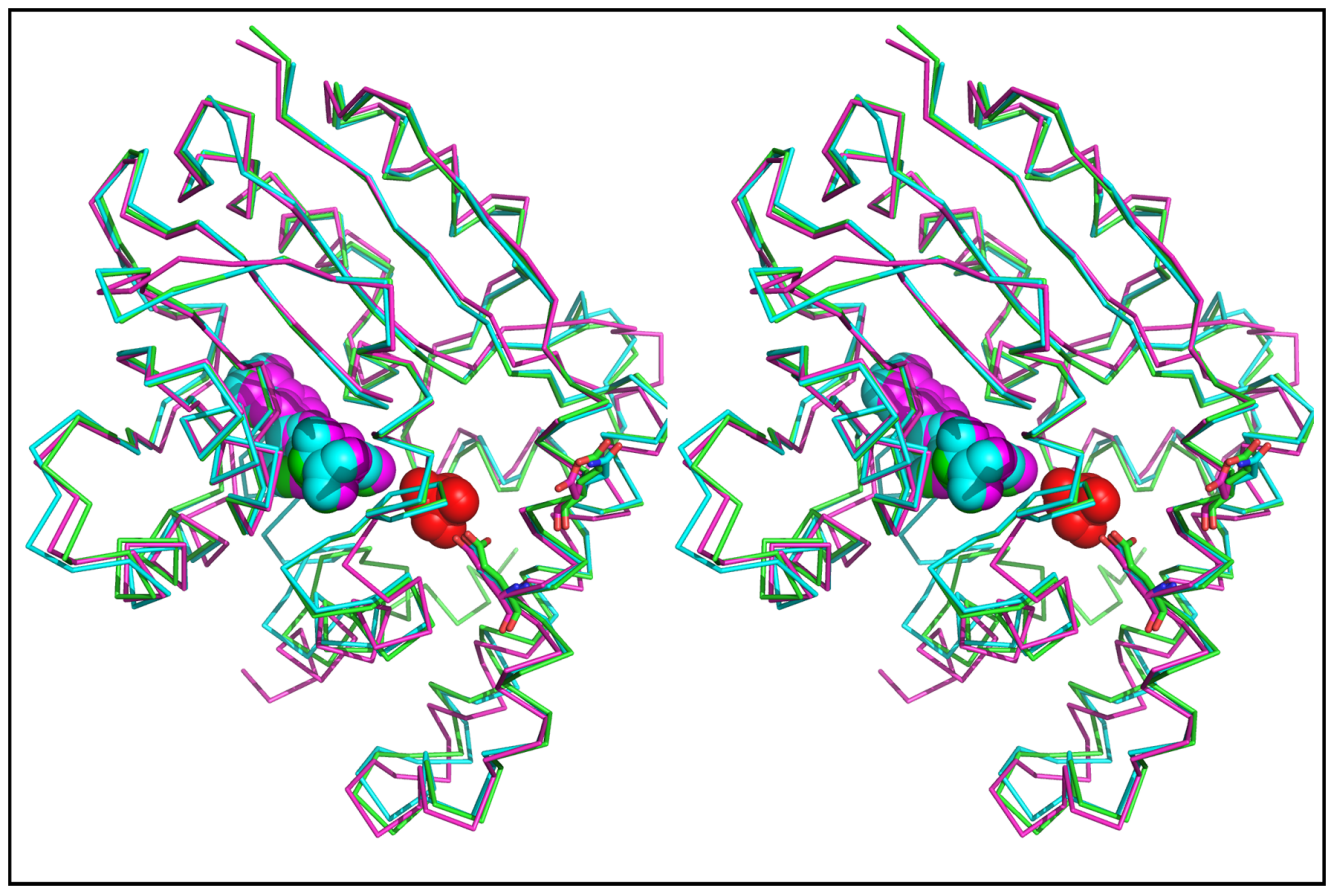
Stereo representation of a pairwise alignment of PvPMT:SAM:PO4 (green, PDB code: 4IV0) and PkPMT:SAM (pink, PDB code: 4IV8) with PfPMT:SAH:PO4 (cyan, PDB code: 3UJ6) RMSD = 0.452 Å. Proteins represented as ribbon Cα trace, SAM and PO4 as spheres. The AQ binding site and interacting residues based on crystal structure reported here are in supplemental figures 3 and 4.

A.G.: Performed the in vitro biochemical assays, contributed to the writing of the main manuscript text and prepared figures 1, 2 and 9; T.L.: Solved the structures of PvPMT and PkPMT and prepared figures 5, 6 and 7; V.K.: Helped with the analysis of the structures, contributed to the writing of the main manuscript text and prepared figures 5, 6 7 and 8; J.C.: Performed the complementation assays in yeast, contributed to the writing of the main manuscript text and prepared figures 3, 4 and 9; Y.A.: designed the codon optimized PkPMT and PvPMT; D.R.V.: Supervised the complementation assays in yeast and contributed to the writing of the main manuscript text; S.N.: Supervised the structural studies and contributed to the writing of the main manuscript text; C.B.M.: Designed the project, supervised the biochemical and functional assays and contributed to the writing of the main manuscript text. All authors reviewed the manuscript.

## Supplementary Material

Supplementary Information Supplementary Information

## Figures and Tables

**Table 1:** Kinetic parameters of PkPMT and PvPMT enzymes

Enzyme/Substrate	V_max_ (nmolmin^−1^ mg^−1^Protein)	*K_c_*_at_ (min^−1^)	*K*_m_ (mM)	*K*_cat_/*K*_m_ (min ^−1^mM^−1^)
PvPMT/P-Etn	18.67 ± 2.93	0.05 ± 0.01	0.21 ± 0.07	0.28
PvPMT/SAM	12.62 ± 0.95	0.04 ± 0.02	0.23 ± 0.07	0.17
PkPMT/P-Etn	86.01 ± 8.35	0.26 ± 0.02	0.10 ± 0.03	2.6
PkPMT/SAM	119.7 ± 15.41	0.37 ± 0.03	0.27 ± 0.09	1.37

**Table 2:** Data collection and refinement statistics for PkPMT and PvPMT

	PkPMT	PvPMT	PvPMT – AQ
**Data Collection**			
Space Group	P2_1_2_1_2	P2_1_	P2_1_
a, b, c (Å)	38.5, 96.8 169.7	38.2, 86.9, 79.6	37.4, 86.6, 78.3
a, b, g	90.0, 90.0, 90.0	90, 101.1, 90	90.0, 103.2, 90.0
Resolution (Å)	20–1.9 (1.95–1.9)	20–1.4 (1.44–1.4)	36.4–1.5 (1.54–1.5)
R_sym_(%)	8.9 (74.8)	8.2 (55.6)	9.7 (56.0)
I/s(I)	12.8 (2.6)	13.6 (3.0)	9.8 (2.4)
Completeness (%)	99.8 (100.0)	98.8 (97.4)	99.3 (98.6)
Redundancy	7.1 (7.2)	6.2 (5.9)	4.1 (3.9)
			
**Refinement**			
Resolution (Å)	19.7–1.9	19.7–1.4	36.5–1.5
No. reflections	48,430	93,902	73,262
R_work_/R_free_	23.3/27.1	15.7/17.8	15.2/17.8
**Number of atoms**			
Protein	4062	4385	4321
SAM	54	54	54
Amodiaquine	-	-	25
Water	363	992	727
**B-factors (Å^−2^**)			
Protein	26.3	11.8	14.1
SAM	29.6	8.4	8.1
Amodiaquine	-	-	27.6
Water	35.6	26.3	28.7
**R.M.S. deviation**			
Bond lengths (Å)	0.007	0.006	0.006
Bond angles (°)	1.15	1.14	1.14
			
**PDB Codes**	**4IV8**	**4IV0**	**4MWZ**
