# Supplementary material for: Structure, Function and Inhibition of the Phosphoethanolamine Methyltransferases of the Human Malaria Parasites Plasmodium vivax and Plasmodium knowlesi
Source: Sci Rep. 2015 Mar 12;5:9064. doi: 10.1038/srep09064 (PMC4357015; doi:10.1038/srep09064)

Supplementary Information: Structure, Function and Inhibition of the Phosphoethanolamine Methyltransferases of the Human Malaria Parasites *Plasmodium vivax* and *Plasmodium knowlesi*

Aprajita Garg[1*], Tiit Lukk[2,4*], Vidya Kumar[1*], Jae-Yeon Choi[3], Yoann Augagneur[1], Dennis R. Voelker[3], Satish Nair[2] and Choukri Ben Mamoun[1]

[1]Department of Internal Medicine, Yale University School of Medicine, New Haven CT, 06520 USA. [2]Department of Biochemistry, University of Illinois at Urbana-Champaign. [3]Basic Science Section, Department of Medicine, National Jewish Health, Denver, Colorado 80206. [4]Cornell High Energy Synchrotron Source, Cornell University

*: These authors contributed equally to this paper

To whom correspondence should be addressed: Choukri Ben Mamoun, Department of Medicine, Yale University School of Medicine, 15 York Street, WWW-403D, New Haven, CT, 06510-3221, USA. Tel: 203-737-1972; Fax: 203-737-1972; E-mail: choukri.benmamoun@yale.edu

Supplementary Figure 1: Sequence alignment of PMT enzymes from *P. knowlesi*, *P. vivax* and *P. falciparum*. Sequence identity is represented by * and sequence similarity by **:**. Residues in red represent the sites for phosphoethanolamine binding; those in green for SAM binding and those in blue are those involved in the interaction with AQ.

```
PvPMT              MISEPVDIKYLENNQYSDEGIKAYEFIFGEDYISSGGIIATTKILSDIQLDANSKVL 57
PkPMT              MVSESVDIEYLENNQYSDEGIKAYEFIFGEDYISSGGIVATTKILSDIYLEPNSKVL 57
PfPMT           MTLIENLNSDKTFLENNQYTDEGVKVYEFIFGENYISSGGLEATKKILSDIELNENSKVL 60
                         *  :******:***:*.*******:******: **.****** *: *****

PvPMT           DIGSGLGGGCKYINEKYGAHVHGVDICEKMVTIAKLRNQDKAKIEFEAKDILKKDFPEST 117
PkPMT           DIGSGLGGGCKYINEKYDAHVYGVDICEKMIAIAKLRNKDKSKVEFEAMDILKKDFPECT 117
PfPMT           DIGSGLGGGCMYINEKYGAHTHGIDICSNIVNMANERVSGNNKIIFEANDILTKEFPENN 120
                ********** ******.**.:*:***.::: :*: * ..: *: *** ***.*:*** .

PvPMT           FDMIYSRDSILHLSYADKKMLFEKCYKWLKPNGILLITDYCADKIENWDEEFKAYIKKRK 177
PkPMT           FDMIYSRDAILHLPYADKKKLFEKCYKWLKPNGILLITDYCADKIENWDEEFKAYINKRK 177
PfPMT           FDLIYSRDAILHLSLENKNKLFQKCYKWLKPTGTLLITDYCATEKENWDDEFKEYVKQRK 180
                **:*****:****.  :*: **:********.* ******** : ****:*** *:::**

PvPMT           YTLMPIQEYGDLIKSCKFQNVEAKDISDYWLELLQLELSKLEEKKEEFLKVYSIKEYNSL 237
PkPMT           YTLIPIQDYGDLIKSCNFQNVQAKDISDYWLELLQMELNKLEEKKDEFLKLYPTDEYNSL 237
PfPMT           YTLITVEEYADILTACNFKNVVSKDLSDYWNQLLEVEHKYLHENKEEFLKLFSEKKFISL 240
                ***:.:::*.*::.:*:*:** :**:**** :**::* . *.*:*:****::. .:: **

PvPMT           KDGWTRKIKDTKRDLQKWGYFKAQK- 256
PkPMT           KDGWTRKIKDTKRHLQKWGYFKAQK- 256
PfPMT           DDGWSRKIKDSKRKMQRWGYFKATKN 266
                .***:*****:**.:*:****** *
```

Supplementary Figure 2: Binding of AQ with respect to PO4 and SAM. Blue is SAM/PO4 structure, red is SAM/PO4, CQA structure. Distance between the carboxylate group of SAM and the hydroxyl group of the phenyl ring of amodiaquine is 11.7 Å.

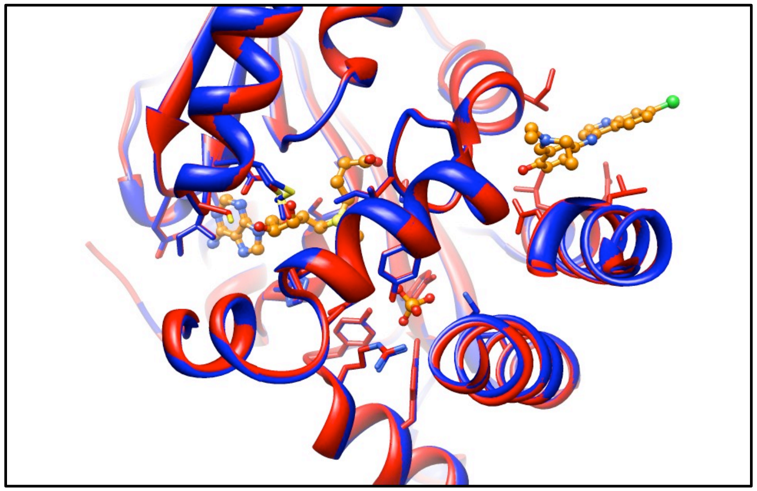

Supplementary Figure 3: Depiction of B chain of the asymmetric unit with its symmetry related *B neighbor. Notice the 2Å translation of the symmetry related molecule. (PvPMT–SAM-PO4 structure in blue; PvPMT-SAM-PO4-AQ structure in red).

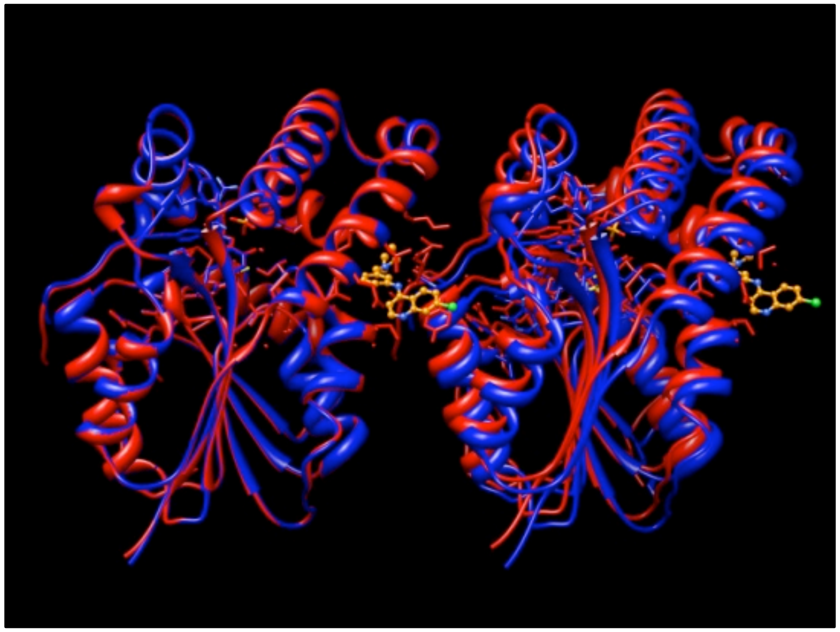

Supplementary Figure 4: Stereo view of the secondary binding pocket of AQ in PfPMT, bottom the same site in PvPMT.

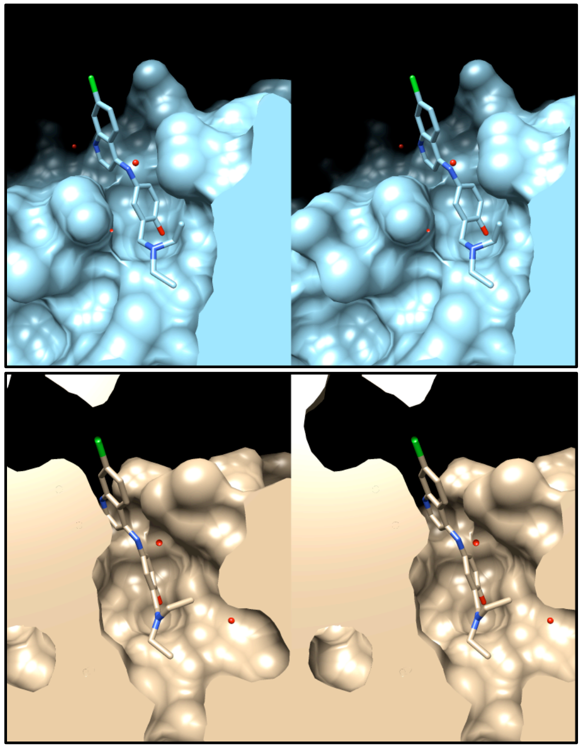

Supplementary Figure 5: Pairwise alignment of PvPMT:AQ:SAM:PO4 (protein in green, AQ (yellow), SAM (gray) and PO4 (red) in stick model; PDB code: 4MWZ) with PfPMT:AQ:SAH:PO4 (protein in cyan, AQ (tan) in stick model; PDB code: 4FGZ).

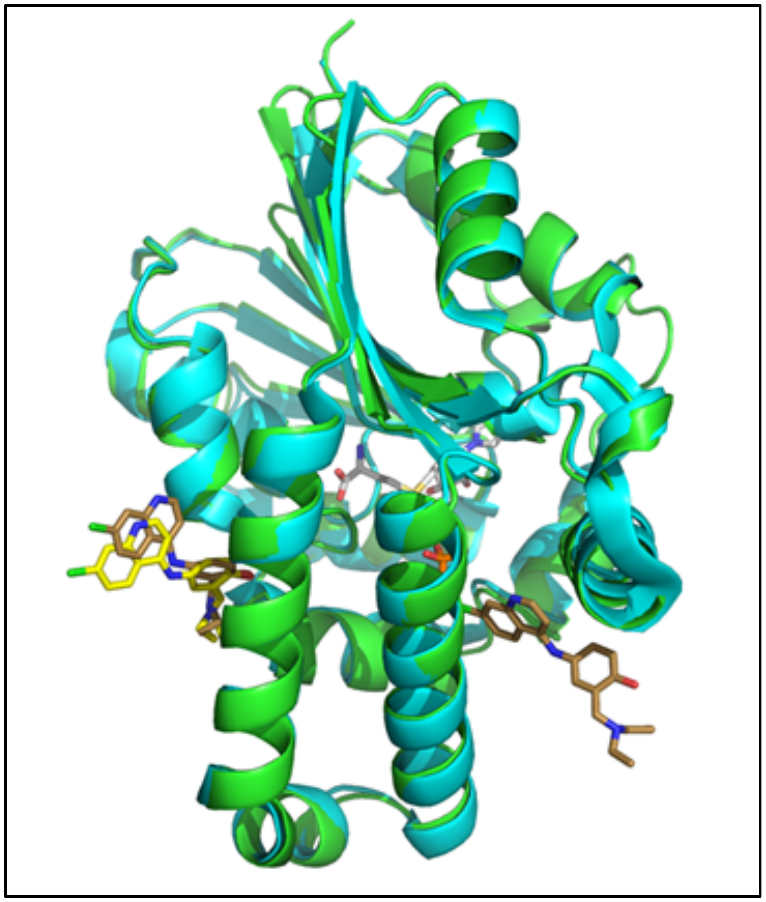

Supplement: Supplementary Information [file srep09064-s1.pdf]
